# Health care utilisation two years prior to suicide in Sweden: a retrospective explorative study based on medical records

**DOI:** 10.1186/s12913-022-08044-9

**Published:** 2022-05-17

**Authors:** Erik Bergqvist, Sara Probert-Lindström, Elin Fröding, Nina Palmqvist-Öberg, Anna Ehnvall, Charlotta Sunnqvist, Tabita Sellin, Marjan Vaez, Margda Waern, Åsa Westrin

**Affiliations:** 1 Department of Clinical Sciences Lund, Psychiatry, Lund University, Baravägen 1, 221 85 Lund, Sweden; 2 Psychiatric In-Patient Clinic, Hallands Sjukhus Varberg, Region Halland, 432 81 Varberg, Sweden; 3 Office of Psychiatry and Habilitation, Region Skåne, 221 85 Lund, Sweden; 4 School of Health and Welfare, The Jönköping Academy for Improvement of Health and Welfare, Jönköping University, 551 11 Jönköping, Sweden; 5 Region Jonköpings Län, Jönköping, Sweden; 6 Department of Psychiatry and Neurochemistry, Institute of Neuroscience and Physiology, University of Gothenburg, 413 45 Gothenburg, Sweden; 7 Psychiatric Out-Patient Clinic, Region Halland, 432 43 Varberg, Sweden; 8 Faculty of Health and Society, Department of Care Science, Malmö University, 214 28 Malmö, Sweden; 9 Faculty of Medicine and Health, University Health Care Research Center, Örebro University, 701 82 Örebro, Sweden; 10 Division of Insurance Medicine, Department of Clinical Neuroscience, Karolinska Institutet, 171 77 Stockholm, Sweden; 11 Psychosis Clinic, Sahlgrenska University Hospital, Region Västra Götaland, 431 30 Mölndal, Sweden

**Keywords:** Suicide, Health services, Health care utilisation, Medical records

## Abstract

**Objective:**

Previous literature has suggested that identifying putative differences in health care seeking patterns before death by suicide depending on age and gender may facilitate more targeted suicide preventive approaches. The aim of this study is to map health care utilisation among individuals in the two years prior to suicide in Sweden in 2015 and to examine possible age and gender differences.

**Methods:**

Design: A retrospective explorative study with a medical record review covering the two years preceding suicide.

Setting**:** All health care units located in 20 of Sweden’s 21 regions.

Participants**:** All individuals residing in participating regions who died by suicide during 2015 (*n* = 949).

**Results:**

Almost 74% were in contact with a health care provider during the 3 months prior to suicide, and 60% within 4 weeks. Overall health care utilisation during the last month of life did not differ between age groups. However, a higher proportion of younger individuals (< 65 years) were in contact with psychiatric services, and a higher proportion of older individuals (≥ 65 years) were in contact with primary and specialised somatic health care. The proportion of women with any type of health care contact during the observation period was larger than the corresponding proportion of men, although no gender difference was found among primary and specialised somatic health care users within four weeks and three months respectively prior to suicide.

**Conclusion:**

Care utilisation before suicide varied by gender and age. Female suicide decedents seem to utilise health care to a larger extent than male decedents in the two years preceding death, except for the non-psychiatric services in closer proximity to death. Older adults seem to predominantly use non-psychiatric services, while younger individuals seek psychiatric services to a larger extent.

## Introduction

In 2008, the Swedish government passed a public health bill with a Vision Zero initiative for suicide prevention [1]. The overall ambition of the legislation was that "No individual should find him- or herself in a situation in which they experience that the only solution is suicide". Yet about 1500 individuals, 1000 males and 500 females, die by suicide each year in Sweden. This includes approximately 1200 deaths with clear evidence of suicide intent (certain suicides); the others are deaths of undetermined intent (uncertain suicides) [2].

Almost all mental disorders are associated with an increased risk of future suicide [3–5]. In many countries, including Sweden, mental illnesses such as depression, anxiety and substance abuse are primarily diagnosed and treated in primary health care and referred to psychiatric services when needed. Additionally, as stated in previous international research, many suicidal individuals are in treatment at primary health care before death by suicide. For example, a systematic review from 2002 covering studies mainly from the United Kingdom and the USA presented an approximate contact rate to primary health care within one month before death by suicide of 45% (range = 20–76%), while the approximate contact rate to psychiatric services in the same period before suicide was 19% (range = 7–28%) [6]. A similar, more recent systematic review covering 15 countries in Europe, North America, Asia and Australia showed comparable utilisation rates [7], as well as a meta-analysis covering psychiatric utilisation before suicide in primarily Western European and North American countries [8]. In addition, a large longitudinal study from the USA of over 5000 individuals who died by suicide from 2000 to 2010 reported that 84% had a health care contact within one year before suicide, but mainly with primary or specialised somatic secondary health care [9]. A population-based case-controlled study from Wales, including all suicide cases 2000–2017, showed that 85% had contact with a general practice within one year, 68% within one month, and 26% within one week prior to death by suicide [10]. A similar Norwegian study, comprising all suicide cases from 2006–2015, presented a higher consultation rate with general practitioners among female (89%) than male (80%) suicide decedents within one year before death [11].

Few studies have focused on the health care utilisation prior to suicide in Sweden [12, 13]. In the general population, about 70–80% of all seek primary care at least once a year [14] and 5.2% of all citizens over 18 had contact with psychiatric services in 2020 [15]. A register-based study showed that 23% of male and 31% of female individuals who died by suicide in Sweden between 1991–2003 had been hospitalised with a psychiatric disorder during the year that preceded death, and 3% of male and 5% of female deaths had occurred either during hospitalisation or on the day after discharge [16].

Still, there is a lack of knowledge of overall health care utilisation, including inpatient and outpatient care, prior to death by suicide in Sweden. Since Sweden has a mainly decentralised and publicly funded health care system [17] that differs from many of the countries previously investigated, the health care utilisation prior to suicide may be different in a Swedish context. Prior international studies state that older adults are less likely to attend psychiatric services than younger [18, 19], and the male utilisation of psychiatric services is lower than female utilisation [20, 21]. However, it is unknown if there are any age or gender differences regarding health care contacts before death by suicide in Sweden. Although health care utilisation in individuals who later died by suicide may reflect overall health care seeking utilization in general [22], previous literature [6, 23, 24] has suggested that identifying putative differences in health care seeking patterns before death by suicide depending on age and gender may facilitate more targeted suicide preventive approaches.

### Aim of the study

The overall aim of this study is to map health care utilisation among individuals in the two years prior to suicide in Sweden in 2015 and to examine possible age and gender differences.

## Method and material

We carried out a retrospective explorative study based on data collected from medical records of people who died by suicide in Sweden during 2015.

The present study was carried out within the ongoing nationwide research project titled Retrospective investigation of health care utilisation of individuals who died by suicide in Sweden 2015. The project includes data for 20 of Sweden’s 21 regions (Stockholm data not currently available).

### Study population

Using the Swedish Cause of Death Register [25] (held by the National Board of Health and Welfare), which comprises data on all dates and causes of death of residents in Sweden, identification of all individuals recorded with death by suicide from 1^st^ of January 2015 to the 31 December 2015 was possible. In this study, only certain suicides were included due to the unclear nature of uncertain suicides and the risk of including deaths that are accidents or due to other reasons of death. Hence, suicide was classified as intentional self-harm, coded as X60-X84 by the International Classification of Diseases, 10th revision (ICD-10) [26]. Individuals were identified by the unique personal identification number assigned to all residents in Sweden. Each individual in the dataset was given a specific code for de-identification. The total number of certain suicides in Sweden in 2015 was 1186. Among those, 232 were excluded because data from Region Stockholm were not available at the time of this study. In all, 949 individuals were identified as the study population. One was excluded due to a pre-existing confidentiality agreement. Five additional suicides were later registered in connection with an update of the Swedish Cause of Death Register; these were not included.

### Data collection

Health care in Sweden is decentralised and managed by regional councils in each county. After establishing a confidentiality agreement based on the Swedish Law of Patient Confidentiality [27] with a representative in each county, personal identification numbers of individuals who died by suicide in 2015 in the specific county and corresponding de-identification codes were sent to the representative by registered post.

Medical records were obtained for the individuals with a health care contact within two years prior to death by suicide, most often by granting access to the regional electronic health record system. Some regions sent paper copies.

### Medical record investigation protocol

This study's definition of health care contact was either a visit or a phone call to any health care provider noted in the medical record in any health care setting (including inpatient care, outpatient care, mobile teams).

A structured investigation protocol was used to review each patient’s medical records. The research group developed the investigation protocol based on guidelines from the Swedish Psychiatric Organisation [28] and specific regional suicide prevention protocols [29]. The investigation protocol contained questions regarding health care utilisation in psychiatric services, primary health care and specialised somatic health care. Furthermore, questions about contact with authorities, psychosocial problems and post-suicide reports from health care providers to the supervisory authority were also included in the protocol.

Medical records from psychiatric services, primary health care and specialised somatic health care dated from death and the previous 24 months were reviewed. All data were compiled into a master file for statistical analysis. The medical records were reviewed during 2016–2020 in all included counties.

### Medical record reviewers

In each county, health care professionals were engaged as record reviewers after signing a confidentiality agreement with the representative in the county. The reviewers were of different professions within health care. However, all were familiar with the electronic health record system and its terminology.

All reviewers were given training sessions in using the investigation protocol. The training was held by members of the research group and was offered on several occasions since new reviewers were included continuously. After the training, the reviewers had access to continuous support and updates by the research group. In addition, a written guide was created to further assist reviewers in data collection. If a reviewer in the data collection came across an individual they had met as a clinician; someone else would investigate the records of this individual in order to maintain objectivity.

### Data analysis

The data were analysed by health care settings (primary care, psychiatric services, and specialised somatic health care). Firstly, descriptive analysis was used to estimate the proportion of individuals having a health care contact within different observation periods: 24 months, 12 months, three months, four weeks, one week and one day before death. Secondly, differences in health care contacts regarding age (0–24, 25–44, 45–64, 65 +) and gender (male, female) were tested using chi2 analysis. Finally, the median time from last health care contact and death was compared regarding gender using the Mann–Whitney U test. Kruskal Wallis H test was used to compare age groups and the median time between last heath care contact and death. As per convention, results were considered statistically significant when two-sided *p* < 0.05. All analyses were carried out in IBM SPSS® version 26 [30] and 27 [31].

## Results

Among the 948 included individuals (681 men, 257 women), the mean age was 51.65 years (SD: 19.12). Characteristics of the study population are presented in Table 1, including marital and occupation status and the proportion on sick leave. There were more people living alone than living with a partner or spouse. The level of unemployment was inferior to the level of some regular activity (employment/school/work training) including age pension. About one fifth were on sick leave at the time of death.

**Table 1:** Characteristics of the study population

	**Total N (%, column)**	**Male N (%, row)**	**Female N (%, row)**
**Total**	948 (100)	691 (72.9)	257 (27.1)
**Age categories (years)**			
0–24	86 (9.1)	56 (65.1)	30 (34.9)
25–44	254 (26.8)	183 (72.0)	71 (28.0)
45–64	352 (37.1)	266 (75.6)	86 (24.4)
65 +	256 (27.0)	186 (72.7)	70 (27.3)
	**Total N (%, column)**	**Male N (%, column)**	**Female N (%, column)**
**Marital status**			
Married/cohabitant	260 (27.4)	195 (28.2)	65 (25.3)
Unmarried/divorced/widower	411 (43.4)	286 (41.4)	125 (48.6)
Unknown status	277 (29.2)	210 (30.4)	67 (26.1)
**Occupation status**			
In employment/school/work training/age pension	507 (53.5)	344 (49.8)	163 (63.4)
In unemployment	139 (14.7)	103 (14.9)	36 (14.0)
Unknown status	302 (31.9)	244 (35.3)	58 (22.6)
**Sick Leave**			
Yes	201 (21.2)	132 (19.1)	69 (26.8)
No	441 (46.5)	309 (44.7)	132 (51.4)
Unknown status	306 (32.3)	250 (36.2)	56 (21.8)

### Contact with health care two years prior to suicide

**Fig. 1:**
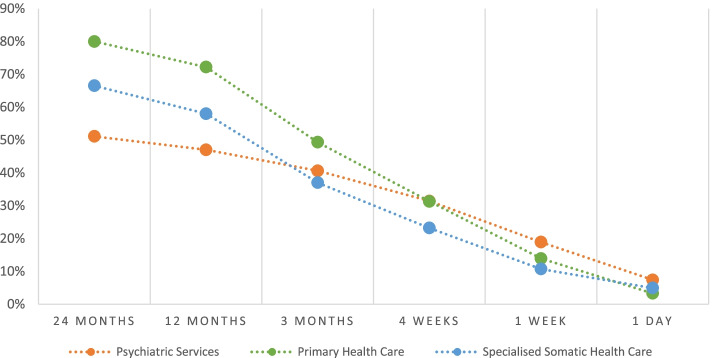
Total health care utilisation within 24 months to 1 day before death

As shown in Fig. 1, the most common type of health care contact within two years to three months before suicide was primary health care. The utilisation rate of primary health care and psychiatric services was the same (31%) within the four weeks prior to death, while psychiatric services were the most common type of health care within one week to one day before suicide.

#### All health care

As shown in Table 2, over 90% of the study population had been in contact with a health care provider during the 24 months prior to suicide, almost 74% within three months and nearly 60% utilised health care within four weeks before suicide. Contact with any health care provider was more common in women than men. Older individuals (≥ 65 years) were in contact with any health care to a greater extent than younger within the 24 months, 12 months and three months observation periods. No age group differences were identified from one month to one day prior to suicide.

**Table 2:** Distribution of the health care utilisation two years prior to suicide

**All health care**												
**Within**	**24 months**		**12 months**		**3 months**		**4 weeks**		**1 week**		**1 day**	
	**N (%)**	***p***	**N (%)**	***p***	**N (%)**	***p***	**N (%)**	***p***	**N (%)**	***p***	**N (%)**	***p***
**Total**	856 (90.3)	-	816 (86.1)	-	698 (73.6)	-	565 (59.6)	-	338 (35.7)	-	137 (14.5)	-
**Sex**												
Male	611 (88.4^a^)	0.001^c^	576 (83.4^a^)	< 0.001^c^	488 (70.6^a^)	0.001^c^	389 (56.3^a^)	0.001^c^	226 (32.7^a^)	0.002^c^	86 (12.4^a^)	0.004^c^
Female	245 (95.3^a^)		240 (93.4^a^)		210 (81.7^a^)		176 (68.5^a^)		112 (43.6^a^)		51 (19.8^a^)	
**Age categories**												
0–24	76 (88.4^b^)	0.031^c^	69 (80.2^b^)	0.001^c^	56 (65.1^b^)	0.007^c^	46 (53.3^b^)	0.261^c^	26 (30.2^b^)	0.739^c^	10 (11.6^b^)	0.326^c^
25–44	223 (87.8^b^)		209 (82.3^b^)		183 (72.0^b^)		152 (59.8^b^)		91 (35.8^b^)		44 (17.3^b^)	
45–64	314 (89.2^b^)		300 (85.2^b^)		251 (71.3^b^)		203 (57.7^b^)		127 (36.1^b^)		44 (12.5^b^)	
65 +	243 (94.9^b^)		238 (93.0^b^)		208 (81.3^b^)		164 (64.1^b^)		94 (36.7^b^)		39 (15.2^b^)	
**Psychiatric services**												
**Within**	**24 months**		**12 months**		**3 months**		**4 weeks**		**1 week**		**1 day**	
	**N (%)**	***p***	**N (%)**	***p***	**N (%)**	***p***	**N (%)**	***p***	**N (%)**	***p***	**N (%)**	***p***
**Total**	484 (51.1)	-	446 (47.0)	-	385 (40.6)	-	298 (31.4)	-	179 (18.9)	-	70 (7.4)	**-**
**Sex**												
Male	316 (45.7^a^)	< 0.001^c^	292 (42.3^a^)	< 0.001^c^	250 (36.2^a^)	< 0.001^c^	190 (27.5^a^)	< 0.001^c^	111 (16.1^a^)	< 0.001^c^	42 (6.1^a^)	0.012^c^
Female	168 (65.4^a^)		154 (59.9^a^)		135 (52.5^a^)		108 (42.0^a^)		68 (26.5^a^)		28 (10.9^a)^	
**Age categories**												
0–24	51 (59.3^b^)	< 0.001^c^	40 (46.5^b^)	< 0.001^c^	36 (41.9^b^)	< 0.001^c^	31 (36.0^b^)	< 0.001^c^	19 (22.1^b^)	0.004^c^	8 (9.3^b^)	0.018^c^
25–44	162 (63.8^b^)		151 (59.4^b^)		131 (51.6^b^)		105 (41.3^b^)		60 (23.6^b^)		28 (11.0^b^)	
45–64	189 (53.4^b^)		177 (50.3^b^)		147 (41.8^b^)		108 (30.7^b^)		70 (19.9^b^)		24 (6.8^b^)	
65 +	82 (32.0^b^)		78 (30.5^b^)		71 (27.7^b^)		54 (21.1^b^)		30 (11.7^b^)		10 (3.9^b^)	
**Primary health care**												
**Within**	**24 months**		**12 months**		**3 months**		**4 weeks**		**1 week**		**1 day**	
	**N (%)**	***p***	**N (%)**	***p***	**N (%)**	***p***	**N (%)**	***p***	**N (%)**	***p***	**N (%)**	***p***
**Total**	758 (80.0)	-	684 (72.2)	-	467 (49.3)	-	295 (31.3)	-	132 (13.9)	-	31 (3.3)	-
**Sex**												
Male	539 (78.0^a^)	0.014^c^	483 (69.9^a^)	0.011^c^	322 (46.4^a^)	0.007^c^	209 (30.2^a^)	0.342^c^	88 (12.7^a^)	0.083^c^	19 (2.7^a^)	0.140^c^
Female	219 (85.2^a^)		201 (78.2^a^)		145 (56.4^a^)		86 (33.5^a^)		44 (17.1^a^)		12 (4.7^a^)	
**Age categories**												
0–24	63 (73.3^b^)	< 0.001^c^	52 (60.5^b^)	< 0.001^c^	26 (30.2^b^)	< 0.001^c^	10 (11.6^b^)	< 0.001^c^	6 (7.0^b^)	0.001^c^	1 (1.2^b^)	0.112^c^
25–44	182 (71.7^b^)		151 (59.4^b^)		91 (35.8^b^)		58 (22.8^b^)		23 (9.1^b^)		6 (2.4^b^)	
45–64	286 (81.3^b^)		263 (74.7^b^)		180 (51.1^b^)		115 (32.7^b^)		53 (15.1^b^)		10 (2.8^b^)	
65 +	227 (88.7^b^)		218 (85.2^b^)		170 (66.4^b^)		112 (43.8^b^)		50 (19.5^b^)		14 (5.5^b^)	
**Specialised somatic health care**												
**Within**	**24 months**		**12 months**		**3 months**		**4 weeks**		**1 week**		**1 day**	
	**N (%)**	***p***	**N (%)**	***p***	**N (%)**	***p***	**N (%)**	***p***	**N (%)**	***p***	**N (%)**	***p***
**Total**	630 (66.5)	-	550 (58.0)	-	351 (37.0)	-	220 (23.2)	-	101 (10.7)	-	46 (4.9)	-
**Sex**												
Male	437 (63.2^a^)	0.001^c^	384 (55.6^a^)	0.012^c^	247 (35.7^a^)	0.181^c^	152 (22.0^a^)	0.148^c^	73 (10.6^a^)	0.883^c^	33 (4.8^a^)	0.857^c^
Female	193 (75.1^a^)		166 (64.6^a^)		104 (40.5^a^)		68 (26.5^a^)		28 (10.9^a^)		13 (5.1^a^)	
**Age categories**												
0–24	48 (55.8^b^)	< 0.001^c^	40 (46.5^b^)	< 0.001^c^	21 (24.4^b^)	< 0.001^c^	16 (18.6^b^)	< 0.001^c^	5 (5.8^b^)	0.015^c^	3 (3.5^b^)	0.648^c^
25–44	142 (55.9^b^)		117 (46.1^b^)		63 (24.8^b^)		41 (16.1^b^)		18 (7.1^b^)		11 (4.3^b^)	
45–64	232 (65.9^b^)		199 (56.5^b^)		126 (35.8^b^)		75 (21.3^b^)		40 (11.4^b^)		16 (4.5^b^)	
65 +	208 (81.3^b^)		194 (75.8^b^)		141 (55.1^b^)		88 (34.4^b^)		38 (14.8^b^)		16 (6.3^b^)	

^a^% within sex

^b^% within the age category

^c^Pearson Chi-Square (2-sided)

#### Psychiatric services

Table 2 shows that slightly over half was in contact with psychiatric services within the 24-month observation period and almost 41% within three months. Almost one-third was in contact with psychiatric services within the four weeks prior to suicide, almost one-fifth during the preceding week and less than one-tenth within one day before suicide. The proportion of women in contact with psychiatric services was higher than among men, and this difference remained for all observation periods.

A larger proportion of younger individuals (< 65 years) was in contact with psychiatric services compared to older individuals (≥ 65 years). For example, nearly a quarter of all individuals aged 25 to 44 years had a psychiatric contact within one week before suicide. Twelve per cent of individuals over 65 years had a similar contact.

#### Primary health care

Eighty per cent of the study population were in contact with primary health care within 24 months, almost half of all within three months and nearly a third were in contact with primary health care within four weeks before suicide. Women were in contact with primary health care more often than men within the 24 months, 12 month and three-month observation period. No gender differences were found closer to suicide. Older individuals (≥ 65 years) were in contact with primary health care to a greater extent than younger individuals (< 65 years) within all observation periods except the day before suicide.

#### Specialised somatic health care

Two-thirds were in contact with any specialised somatic health care within 24 months before suicide and nearly two-fifths within three months. Almost one-fourth were in contact with specialised somatic health care within four weeks and slightly over one in ten within one week before suicide. Paralleling primary care contacts, a larger proportion of women was in contact with somatic health care than men within 24 months and 12 months of observation. However, there was no difference between men and women closer to suicide. Likewise, older individuals (≥ 65 years) were in contact with specialised somatic health care to a greater extent than younger individuals (< 65 years) within all observation periods, except within one day.

### Time since last health care contact

Among those with any health care contact within two years before suicide (*n* = 856), the median time between the last health care contact and death by suicide was 12 days (Interquartile range (IQR) 3 to 53). Among those in contact with psychiatric services (*n* = 484), the corresponding figures between the last contact and suicide were 16 (IQR 4 to 60) days. For those in contact with primary care (*n* = 758), the median time between the last contact in primary health care and suicide was 51 (IQR 12 to 165) days. Finally, for those in contact with specialised somatic care (*n* = 630), the median time between the last contact in specialised somatic care and suicide was 70 days (IQR 16 to 223).

Overall, women had a lower median time between last health care contact (any) and suicide (women: 9 days, men: 14 days. Mann Whitney U test: *p* = 0.002), but no gender differences were identified when analysing psychiatric services, primary care and specialised somatic care separately.

No difference between age groups was found when analysing median time between last health care contact and suicide in the whole group who were in contact with any health care or the subgroup of individuals who had been in contact with psychiatric services. However, older adults had a significantly shorter period between last non-psychiatric health care contact to suicide than younger people (Primary health care: age 0–24: 90 days, age 25–44: 90 days, age 45–64: 48.5 days, age 65 + : 30 days. Kruskal Wallis Test: *p* < 0.001); (Specialised somatic health care: age 0–24: 156.5 days, age 25–44: 107.5 days, age 45–64: 72 days, age 65 + : 40.5 days, Kruskal Wallis Test: *p* < 0.001).

## Discussion

Our study shows that over 90% of the study population was in contact with a health care provider during the 24 months prior to suicide, almost 74% within three months and nearly 60% had such contact during the last four weeks. Moreover, half of the care utilisers had a health care contact within 12 days and a quarter within three days prior to suicide. Visits to primary health care were the most common type of care contact during the observation period, except for the final four weeks of life, where psychiatric care utilisation was more prevalent, although the utilisation varied by gender and age. The characteristics of the study population varied. The number of participants in a single household was for example larger than those living with a partner or a spouse, although the rate of unknown status was high due to lack of data.

Within twelve months before death, 86% had a health care contact, and proportions with primary health care contacts and specialised somatic health care contacts were greater than the proportion with health care contacts at psychiatric services. This is in line with the results presented by Ahmedani et al. [9], who reported that 84% had a health care contact within twelve months before suicide, but mainly with primary or specialised somatic secondary health care.

As to primary health care utilisation, 72% were in contact within twelve months, 31% within one month and 14% within one week before death by suicide. The results show a slightly lower consultation rate than corresponding numbers of Stene-Larsen et al. [7], Louma et al. [6], Hauge et al. [11] and John et al. [10]. Hence, the primary health care utilisation before suicide in Sweden seems to be a bit lower than in the other investigated countries. However, the utilisation of primary health care before suicide was higher among Swedish suicide decedents than during the 1980’s [32].

Regarding psychiatric health care, 47% utilised such services within twelve months, 31% within one month and 19% within one week before death by suicide. These are higher contact rates than the average contact rates reported in the review article by Stene-Larsen et al. [7] and the meta-analysis by Valby et al. [8], illustrating a potentially greater utilisation of psychiatric services before suicide in Sweden than in the investigated countries in prior studies. The divergent prevalence of psychiatric utilisation could be due to different types of health care organisations in various countries. Proportions in contact with psychiatric services in our study were similar to those reported in a register-based study from Norway [33], which has a similar publicly financed health care system as Sweden.

Women were more often in contact with health care in general. The present study also elucidates a gender difference regarding contact with psychiatric services before suicide, where women were more likely to attend psychiatric services than men. Nevertheless, there was no gender difference regarding contacts with either primary health care or specialised somatic health care in close in time to the death by suicide. Depressive disorders are more prevalent in women than men, especially in adolescents and younger adulthood [34], which could explain the gender differences in care utilisation. Moreover, men with depression may be less likely identified in health care than women due to different symptoms [35], resulting in inadequate diagnosis and undertreatment of male depression. Men experiencing depression are more likely than women to exhibit symptoms of irritability, anger, substance misuse, risk-taking and impulsivity that could mask more typical internalising depressive symptoms [36]. This is compatible with a prior older Swedish study where an educational program in depression diagnosis and treatment to primary care practitioners on the island of Gotland mainly affected the suicide rates of women [37]. Another explanation for the gender differences could be the higher alcohol use disorder (AUD) rates among men [38]. Although AUD is a potent risk factor for completed suicide in both women and men [39], alcohol-related problems are significantly more prevalent among men [38]. Men with AUD are also more likely to develop secondary depressive disorders than women with AUD [40]. Men are also more affected by the stigma associated with mental health services and are more prone to utilise health services not exclusively related to mental health [41].

This study also showed a minor but statistically significant difference between age groups. Older individuals had a higher contact rate than younger with any health care provider within the two years preceding suicide. This could be explained by considerable general morbidity, leading to increased health care utilisation among older adults. Closer in time to death by suicide, within four weeks before suicide, there was no age group difference regarding any health care contacts. However, there were substantial age group differences within four weeks before suicide when analysing different health care services separately. Younger individuals were to a greater extent in contact with psychiatric services than older individuals. The pattern was inverted in contact with primary health care and specialised somatic health care. These findings are in accordance with the study by Louma et al. [6], which showed similar age-related variations in contacts with psychiatric services and primary health care services (within one year and one month before suicide, respectively). One way of understanding the observed phenomenon is that older individuals with depression or other psychiatric disorders may mainly attend primary health care or non-psychiatric specialist care when seeking help. Prior studies indicate that older adults with depressive disorders prefer to use the general health care system rather than mental health clinics [42]. Regardless, this finding suggests a need for geropsychiatric competence within these units, including suicide prevention programs suited for older patients. A recently published cohort study among suicide attempters at psychiatric emergency departments showed a lower prevalence of psychiatric disorders and a lower rate of symptomatology among older adults than young adults, even though the suicidal intent was higher among older than young adults [43]. Further research is required to increase understanding of this issue, including analysing which symptoms are presented in health care among the different age groups before death by suicide. Possibly, older adults are less likely to present symptoms of psychopathology prior to suicide than younger adults, which could explain the age group differences in the type of care utilisation before death.

## Conclusion

This article depicts a health care utilisation before suicide in Sweden that vary by gender and age. The overall health care utilisation in other countries seems to be comparable to Sweden, although the utilisation of primary health care seems to be lower and the utilisation of psychiatric services seems to be higher in Sweden in comparison to other countries. Since many suicide decedents utilise health care shortly before death, our study highlights the importance of suicide prevention strategies within Swedish health care. Currently, suicide prevention interventions are mainly established in psychiatric health services in Sweden. As the rate of psychiatric service use before suicide is relatively high in Sweden, this study shows a need to improve existing prevention strategies (or increase the compliance to the strategies) within psychiatric services. However, this study also indicates a need to implement suicide prevention strategies within all Swedish health care settings. Furthermore, specific strategies for older [44, 45] and male patients [46] may aid suicide prevention efforts in non-psychiatric health care settings.

### Strengths and limitations

The study population provided nearly national coverage of suicide among individuals with all types of health care contacts. A limitation is that data from the Stockholm region were not yet available. The Stockholm region is the most populated in Sweden; approximately 20% of all suicides occur there.

By collecting data from medical records, the study provides detailed data on contacts with all professions in health care. However, all data in medical records are not systematically reported, resulting in relatively large proportions of missing data for some variables, such as marital status, occupation status, and sick leave. Including information from the National Patient Register would have yielded additional details of inpatient care and visits to physicians in psychiatric services and somatic care to the analysis, but not of visits to other care professionals nor visits to primary care.

Many investigators were involved in data collection. Efforts were made to ensure the investigators collected data uniformly. This included group training, investigator guidelines and a high level of support from the research group. However, no systematic testing of inter-rater reliability (IRR) of the data scoring was performed.

In addition, this study did not access all data from the medical records of all private health caregivers. In 2015 in Sweden (excluding the Stockholm area), the portion of private health care varied greatly between regions. Of out-patient health care, between 0–25% of psychiatric services, 0–63% of primary care, and between 0–19% of specialised somatic care was in private management, while in-patient care varied between 0–12% (including both psychiatric and somatic health care) [47]. In the medical records systems investigated in this study, private health care is not always automatically recorded, and data may therefore have been omitted. In cases where information on private care was available in regional registers or medical records, efforts were made to obtain the medical records from the private clinic. The amount of missing data due to the lack of private care records is unknown. A survey was sent out to the record reviewers regarding experienced investigation problems. Overall, reviewers responded that problems accessing non-governmental health care were minimal, and the effect on the results was presumably negligible.

Another methodological consideration is that criteria for classifying deaths as suicides vary across cited studies. We did not include data on individuals who died due to self-harm events of undetermined intent. According to the Swedish Cause of Death Register, such events (“uncertain suicides”) comprised 24% of the total number of certain and uncertain suicides in Sweden in 2015 [2]. In suicide research, it is important to state whether only certain or also uncertain suicides are included as this has implications for the interpretation of the results. By not including uncertain suicides, some deaths by suicide may have been missed and the number of suicides underestimated.

Our study lacks a population-based comparison group. However, it is known from prior US studies that those who die by suicide have more frequent health care usage before death across all settings compared to a matched control group [9, 48, 49].

Since this study investigates the health care utilisation of suicide decedents seven years ago, questions might arise about the generalisability of the results to current care utilisation of suicidal patients. However, the trends of suicide rates in Sweden have been quite stable over the last two decades. According to the Public Health Agency of Sweden, the suicide rate among men aged 45–64 decreased while among women aged 15–29 increased between 2006 and 2018 [50], but no other statistically significant trend was found. Since there, to our concern, has not been any major changes in the Swedish health care sector during the past seven years, we believe the results of this study correspond with the present state.

### Implications for future research

The present study depicts age and gender differences in health care contacts prior to suicide. In further planned studies, we will explore symptomatology, treatments and actions taken by health care professionals in the different health care settings during the weeks and months prior to patient suicide.

## Data Availability

The datasets generated and analysed during the current study are not yet publicly available. This is because our dataset involves still unpublished data, and since we are planning additional future papers with other aspects of health care utilisation prior to suicide, we are currently unable to share our data openly. However, our data are available from the corresponding author upon reasonable request.

## Abbreviation

IQR: Interquartile range
